# The Differential Effects of the Amount of Training on Sensitivity of Distinct Actions to Reward Devaluation

**DOI:** 10.3390/brainsci11060732

**Published:** 2021-05-31

**Authors:** Maya Bar Or, Oded Klavir

**Affiliations:** 1 School of Psychological Sciences, The University of Haifa, Haifa 3498838, Israel; mayabaror@gmail.com; 2 The Integrated Brain and Behavior Research Center (IBBRC), The University of Haifa, Haifa 3498838, Israel

**Keywords:** action-control, goal-directed, action–outcome, habit, stimulus–response, mice behavior

## Abstract

Shifting between goal-directed and habitual behaviors is essential for daily functioning. An inability to do so is associated with various clinical conditions, such as obsessive–compulsive disorder (OCD). Here we developed a new behavioral model in mice allowing us to produce and examine the development of different behaviors under goal-directed or habitual control. By using overtraining of instrumental associations between two levers and two rewards, and later devaluating one of the rewards, we differentiate and explore the motivational control of behaviors within the task which consequentially promotes what seems like excessive irrational behavior. Using our model, we found that the ability of instrumental behavior, to adapt to a change in the value of a known reward, is a function of practice. Once an instrumental action was practiced extensively it becomes habitual and, thus, under S–R control and could not be amended, not even when resulting in a noxious outcome. However, direct consummatory or Pavlovian actions, such as licking or checking, responds immediately to the change in value. This imbalance could render an instrumental behavior excessive and unresponsive to changes in outcome while the direct change in consumption implies that the change was in fact registered. This could suggest a system that, when out of balance, can create excessive behaviors, not adapting to an acknowledged change.

## 1. Introduction

Behavior can range from mindful, deliberate, and carefully planned actions to sets and sequences of nearly automated habitual actions. In appetitive instrumental learning, behavior during initial training is considered goal-directed which can be described as a set of activities aimed at obtaining an end-state [1], and controlled by an action–outcome (A–O) contingency. However, with extensive training, performance gradually becomes habitual, which is stimulus-driven and automatic [2], and motivated by stimulus–response (S–R) association [3]. Habitual control of actions allows fast and efficient responding to pre-specified stimuli, while changes in circumstances call for re-evaluation and, hence, requires the flexibility that characterizes the goal-directed behavioral control system. The ability to flexibly shift back and forth between habitual and goal-directed control of behaviors is critical for successfully adapting to one’s environment [2], and the failure to do so was suggested to lie at the root of psychopathologies, such as obsessive–compulsive disorder (OCD), obesity, and addictions [2,4,5,6,7,8]. A dysfunction in the processing capabilities of the involved neural circuits could potentially cause information usually processed automatically to intrude into consciousness as obsessions, and cause habits and automated behaviors to become binding as compulsions [9].

In some view, part of the compulsive behaviors demonstrated in OCD might be the consequence of impaired response feedback mechanism, resulting in the inability to adjust behavior to the environment [10,11]. According to this view—compulsions can be characterized as goal-directed activities lacking a proper feedback of action completion. Thus, normal behaviors, such as cleaning or checking, can become compulsory by lacking the proper signal of goal achievement [8]. This view also paved the way for the post training signal attenuation model (PTSA), which is a leading behavioral animal model of compulsive behaviors [12]. In this model, rats press a lever previously associated with a stimulus predicting reward and continue to press it excessively, without checking for reward, after the contingency between the stimulus and the reward has been extinguished. This seemingly excessive and irrational behavior, produced by a mere training schedule in naïve rats was shown to be specifically affected by drugs and treatments effective for alleviating compulsive symptoms [13,14,15], suggesting some mechanistic overlap between the learning mechanisms underlying this phenomena and compulsive behavior [16]. The persistence of lever press behavior, together with the reduction in checking behavior, could stem from a difference in the sensitivity to goal value due to a different motivational control mechanism governing each behavior. PTSA could be regarded as a test for the sensitivity of the behavior to outcome revaluation manipulation. Outcome revaluation are usually tasks which include training an animal to perform a specific action to acquire a rewarding outcome. Post-training, the value of the outcome is modified, and the consequences of this manipulation are tested on the previously acquired action. An action sensitive to revaluation could be considered goal-directed. Contrarily, an action that persists despite a reduction in the outcome value, is not under goal-directed control, and is considered to be under a more habitual control mechanism [17,18].

Instrumental lever press behavior was shown to be sensitive to goal devaluation. A rat will lever press less vigorously after creating aversion from the contingent reward by associating it with illness, caused by lithium chloride injection after reward consumption [19]. This suggests that action–outcome association controls lever press behavior. However, after prolonged training in the same paradigm, devaluating the reward does not seem to change the instrumental lever presses [20], suggesting the lever press action is no longer controlled by the value of the goal, as it became habitual [17]. Nonetheless, this effect is harder to achieve in more complicated tasks in which an animal can execute two different actions to obtain two different rewards. In that case, researchers found equivalent or greater sensitivity of extensively trained instrumental responses to reinforcer devaluation, indicating a dominance of goal-directed control [21]. Although aimed at achieving the same goal, the nature of reward approaching behavior is different than lever pressing behavior. It is not defined as instrumental response and is sometimes defined as a measure for Pavlovian behavior [22]. Moreover, when measured in the same task together with instrumental responses or by itself it seems that reward–approach behavior remains sensitive to direct modification in outcome value even after extensive or stimulus–response promoting training [23,24]. This was suggested to be related to the recruitment of different rewards related neural systems controlling those behaviors [24]. The differential effect of prolonged training on the two behavior types and their different sensitivity to motivational systems could perhaps explain the excessive yet rigid lever press behavior reported by the PTSA model. This specific behavior in the PTSA model was shown to respond to drugs [15], treatments [13], and involve neural mechanisms known to be involved in OCD [25,26,27], implying a common underlying mechanism.

In the current research, we establish a new behavioral model for producing either adaptive goal-directed behavior or habitual and inflexible instrumental behavior in mice. By using overtraining of instrumental associations between two levers and two rewards, and later devaluating one of the rewards, we differentiate and explore the motivational control of the two behaviors within the task, which consequentially promotes the excessive and apparently irrational instrumental behavior.

## 2. Materials and Methods

### 2.1. Behavioral Model

#### 2.1.1. General Description

Mice were trained under two distinct conditions: short training (ST) and extended training (ET). During training, mice access to water was limited to 1–2 h per day.

Mice were placed in an operant chamber within sound attenuating boxes (Med-Associates, St Albans, VT) and trained to press either the left or right lever in response to one of two different auditory tones (5-s tones, 5 or 10 kHz, different tone for each lever). Pressing each lever correctly (only when the associated tone was played) was rewarded by one of several rewards (chocolate milk, condensed milk, strawberry–banana juice, or sugared water (10 g/100 mL)), all tested as equally appealing for the mice in a preliminary test in a choice test preformed in the maze. The sound associated with the lever and the reinforcers were kept constant throughout the experiment for each mouse, with counterbalanced groups for the different rewards. Behavioral measures included lever press behavior, checking behavior (measured by a beam breaker as the number of head entries into the reward chamber), and licking behavior (measured as the number of times the mouse’s tongue touched the liquid, by a dual contact lickometer). All measuring devices and the behavioral recordings were provided by Med Associates, Inc. (St. Albans, VT, USA).

#### 2.1.2. Training

All sessions at each stage started with three minutes of habituation, whilst the box was darkened, and no stimulus was activated.

**Reward port training**—on the first two days of the training, mice were placed in the operant box and after habituation followed by the illumination of the house light, reinforcers were introduced to the two ports on the two sides of the chamber (left/right) in random order. Illumination of the reward port was simultaneous to the reinforcers delivery and lasted 20 s, followed by an inter-trial interval (ITI) of 40 s, whilst the box was darkened, and repeated so on for 12 min (total time of the training session is 15 min).

**Lever press training**—in the next stage of the training, after habituation, both levers were extended simultaneously. Mice were trained to lever press for reward and every lever press was reinforced (simultaneously illuminating the reward port for 20 s). Mice proceeded to the full training stage after passing a preset criterion of 20 lever presses or 5 training days.

**Full training**—during this stage, one of the two tones was played. Meanwhile, both levers were extended for 20 s and only the light above the correct lever associated with the tone was turned on in each session. If mice pressed the wrong lever (the one not associated with the tone that was played), or did not press any lever, both levers were pulled in, and the box was darkened for 40 s. If mice pressed the correct lever the reinforcer assigned to that lever was delivered, with a simultaneous illumination of the reward port for 20 s, followed by an ITI of 40 s darkening of the box. The ST group was trained until reaching a success criterion calculated by chi-square difference from chance over three days or until mice complete 18 days of training. The ET group was trained until mice reached the criterion, plus an overtraining period. The minimum length of the overtraining was 10 days, and mice continued the training until reaching a criterion of 70% correct responses in two consecutive days or a maximum of 17 days of overtraining.

**Devaluation**—one day after the final session of either ST or ET, mice were exposed to one of the reinforcers (with counterbalanced groups for each reinforcer) in the operant chamber for 15 min and were then returned to their home cages. Forty minutes later, mice were injected with 0.15M lithium chloride (LiCl, 2% of body weight). Mice were also exposed to the non-devalued reward on altering days followed by a sterile saline injection (2% of body weight) to measure the specificity of the devaluation to the devalued reinforcer. The procedure continued until mice reduced consumption of the devalued reward to under 15% of licks of the first day of exposure or 12 days of injections.

**Test**—the next day after finishing devaluation, mice underwent a testing session, which was conducted in the same manner as the training sessions, only with no reward after lever pressing.

**Revaluation**—one day after the test session mice returned to full training for 5 days with the rewards reinstated.

### 2.2. Statistics

Data were analyzed using a combination of between subject and repeated measure ANOVA followed by Tukey HSD post-hoc tests for specific effects. Statistical significance level was set at α = 0.05. We assumed the data were collected from a population of normal distribution (though this assumption was not directly tested). Due to the difference in the number of mice in each group, we preformed the Levene’s Test for Homogeneity of Variances for each of our ANOVA analyses. In cases of variance homogeneity violation, we used Welch–Satterthwaite degrees of freedom correction for the relevant planned comparisons and corrected the alpha according to the number of comparisons. Data were statistically analyzed using both MATLAB (MathWorks, Portola Valley, CA, USA) and Statistica (StatSoft, Tulsa, OK, USA).

### 2.3. Animals

Twenty-three C57BL/6J (Envigo, Jerusalem, Israel) male mice, 8–9 weeks old, were housed in groups of 2–5 mice per cage on a 12-h light/dark cycle with ad libitum food, and under water deprivation (approximately 17 h each day). All experiments took place during the dark cycle. All experiments were approved by the Haifa Institute Animal Care and Use Committee (IACUC).

## 3. Results

### 3.1. Training and Devaluation

**Figure 1:**
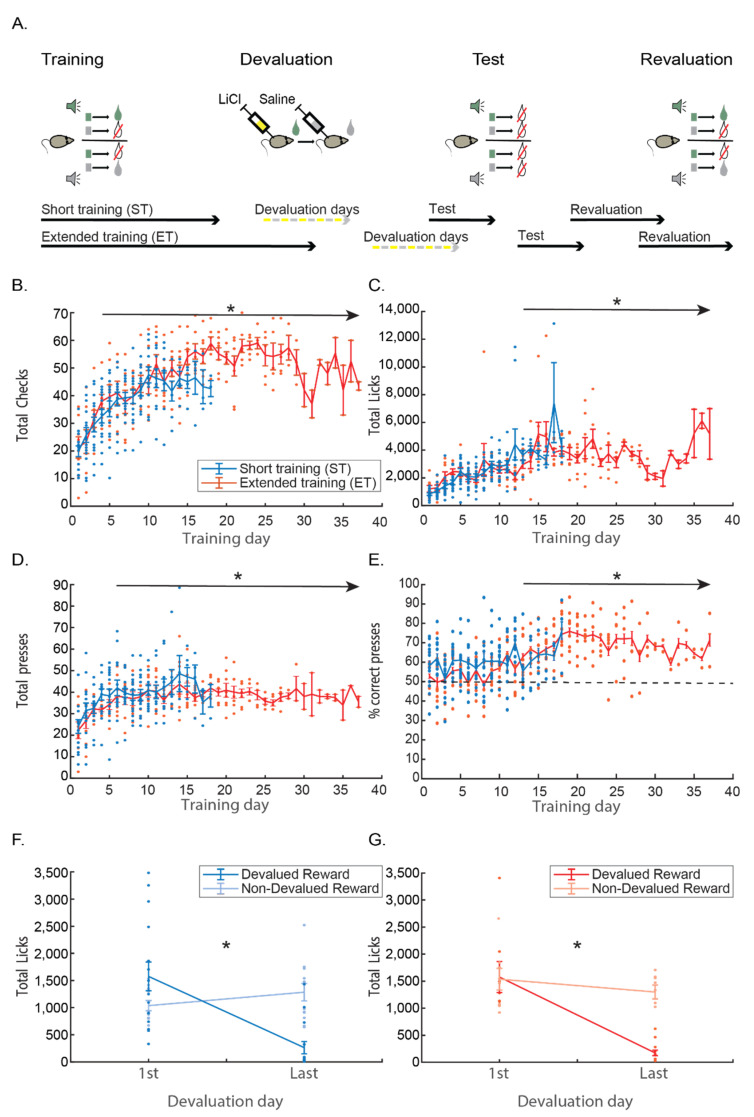
The effects of training on distinctive task behaviors and the following effects of devaluation training. (**A**) Schematic representation of experimental design. In all following figure blue represents the short training group (ST) and red represents the extended training group (ET). Asterisk represents significance of *p* < 0.05 (**B**–**E**) Line plots with error bars (standard error) overlaid with the dots of raw data of each mouse describing the change in total magazine checks (**B**), total licks (**C**), total lever presses (**D**), and total correct lever presses over the course of the task’s training days. (**F**–**G**) Line plots with error bars (standard error) overlaid with the dots of raw data of each mouse describing the change in consumption from the first to the last day of reward devaluation (devaluation included altering days of devalued +LiCl injections and non-devalued rewards + saline injections until reaching learning criterion) for the ST group (**F**) and for the ET group (**G**) both for the devalued reward (darker color) and the non-devalued reward (lighter color).

Mice were randomly divided to the two experimental groups: extended training (ET, *n* = 8) and short training (ST, *n* = 15). Figure 1A shows a scheme of the training timeline for both ET and ST groups. The full details of each stage are in the Materials and Methods section. Shortly: every mouse was trained in all stages of the behavioral paradigm. During training, mice learned to press one of two levers according to a guiding auditory cue, to receive a reward associated to each lever. ST mice were trained until a criterion for task learning was established, while ET mice continued to train long after the criterion was reached. When training was over in both groups, mice entered several consecutive days of devaluation training. Devaluation started with mice consuming one of the rewards followed by LiCl injection thus devaluing that reward. The following day, mice consumed the second reward followed by a saline injection to control for the injection procedure and the specificity of the devaluation procedure. We repeated this order of altering days until devaluation criterion was reached. Following successful devaluation, mice underwent the test, which was conducted in the same manner of the training, but no reward was provided in the reward ports. The last phase was the revaluation phase where mice underwent five days of training with reward again delivered at the ports after correct responding.

#### 3.1.1. Training

Results of the training session for both groups are presented in Figure 1B–E. Average full training duration in the ST group was 13.06 (±0.88) days and 15.37 (±1.13) days in the ET group (not including the over-training period). The average correct response percentage on the day mice reached the success criterion in the ST group was 77.41% (±2.91) and 72.98% (±1.32) in the ET group. ET group overtraining period consisted of an average of additional 11.87 (±1.25) days of training and the average correct response percentage by the end of the overtraining period was 76.11% (±4.34).

We took several behavioral measures to compare throughout the paradigm: checks (the number of head entries into the reward magazine), licks (the number of actual licks on the port), and lever presses. As the ST protocol consists of a significantly smaller number of training days, we used the largest number of comparable days (18 first training days) in order to compare both groups using a two-way ANOVA comparing the training-day X group (ST/ET) for each measure. Training produced a learning curve in all measures, gradually increasing responses above chance level, and maintaining those levels throughout the rest of the training.

Looking at the measure of checks, we found a main effect for training days: F(17, 153) = 17.032, *p* < 0.001, with Tukey HSD post hoc revealing that the number of magazine checks is significantly higher than the beginning of training already on day 4, and that all days from the 4th day onwards are significantly different than the first day (*p* < 0.05; see Figure 1B). For the licking measure, we found a main effect for training days: F(17, 153) = 6.509, *p* < 0.001, with Tukey HSD post hoc revealing that the number of licks is significantly higher than the beginning of training already on day 13, and that all days from the 13th day onwards are significantly different than the first day (*p* < 0.05; Figure 1C). For lever presses, we found a main effect for training days: F(17, 153) = 3.374, *p* < 0.001, with Tukey HSD post hoc revealing that the number of lever presses was significantly higher than the beginning of training already on day 6, and that all days from 6th day onwards were significantly different than the first day (*p* < 0.05; see Figure 1D). From that, it seems that magazine checks and lever presses are quicker to increase in rate as compared to licking.

However, when looking at accumulation of correct lever presses (that is—the percentage of presses on the lever indicated by the tone and leading to reward), learning seems slower. We found a main effect for training days: F(17, 153) = 4.801, *p* < 0.001, with Tukey HSD post hoc revealing that the number of magazine checks was significantly higher than the beginning of training on the 14th day, and that all days from 14th day onwards were significantly different than the first day (*p* < 0.05; see Figure 1E). We also directly compared the number of correct and incorrect presses in that statistical model. A three-way ANOVA—group (ST/ET) * day (1–18 comparable days) * correct/incorrect number of presses revealed a significant main effect for days: F(17, 136) = 2.844, *p* < 0.001. As already reported, we could already see an increase, in general, in lever presses with training from the 6th day training. We also found a main effect for correct presses vs. an incorrect number of presses (F(1, 8) = 15.137, *p* < 0.005) and, most importantly, a day * correct/incorrect interaction (F(17, 136) = 4.0598, *p* < 0.001), showing that, in the early training days, there were no differences, and gradually, as training progresses, we saw more correct than incorrect presses. In fact, Tukey HSD comparisons show that, starting on the 13th day of training, the average difference between the number of correct and incorrect presses becomes significant—meaning towards the end of the ST. While there was a fast increase in general lever pressing at the beginning of the training, by the end of it, the behavior was more precise, and the increase was only in the correct lever presses. It is important to note that no differences in correct vs. incorrect presses were found between the groups.

#### 3.1.2. Devaluation

The success of the devaluation is indicated by the consumption of the devalued reward on the last day of devaluation as compared to the first day. In all, the amount of devaluation days needed to achieve the reduction criterion after training was slightly high, which is expected in this strain of mice [28]. There was no difference between the groups in the average amount of devaluation days to reach criterion or in the actual reduction in licks on the last day of devaluation. ST mice reached criterion within 5.2 devaluation days in average (1.97 STD) and ET mice reached criterion within 4.4 days (1.30 STD). A *t*-test revealed no significant difference between the number of days to reach criterion between the groups (t(21) = 1.061, *p* = 0.3 n.s.). Moreover, ST mice reached criterion of 11.5% licks in average as compared to the first day (13.8 STD) and ET mice reached criterion of 8.9% days (5.89 STD). A *t*-test revealed no significant difference in the average criterion between the groups (t(21) = 0.507, *p* = 0.62 n.s.). The specificity of the devaluation effect to the reward, which was devalued, was indicated by comparing it to the reward that was presented during altering days, but was not devalued. We found that our devaluation procedure was successful in both, as indicated by a decrease in the consumption only of the devalued reward and not the non-devalued reward in both ST and ET groups (Figure 1F–G). When looking at the ST group, a two-way ANOVA with time (first/last day)*condition (devalued/ non-devalued reward) revealed a significant main effect for condition (F(1, 25) = 15.664, *p* < 0.001), indicating more consumption for the non-devalued reward and, most importantly, a time × condition interaction (F(1, 25) = 33.539, *p* < 0.001). Tukey HSD post hoc revealed that consumption on the last day of the devaluation procedure was significantly lower than the first day, only for the devalued reinforcer (*p* < 0.001) but not for the non-devalued (*p* = 0.614; n.s.). When comparing the consumption between the different conditions (devalued and non-devalued reinforcers), we found a significant difference in the consumption of the reinforcers on the last day of exposure (*p* < 0.01), but not the first day (*p* = 0.178; n.s.). These same effects were also true for the ET group, where two-way ANOVA with time (first/last day) × condition (devalued/ non-devalued reward) revealed a significant main effect for condition (F(1, 14) = 7.379, *p* < 0.05), indicating more consumption of the non-devalued reward and, most importantly, a time × condition interaction: F(1, 14) = 11.099, *p* < 0.01. Tukey HSD post hoc showed that the consumption during the last day of devaluation was significantly lower than on the first day, but only for the devalued reinforcer (*p* < 0.001) and not the non-devalued (*p* = 0.77; n.s.). When comparing the consumption between the different conditions (devalued and non-devalued), we found a difference on the last day of exposure (*p* < 0.005), but not the first day (*p* = 0.998; n.s.).

#### 3.1.3. Test

**Figure 2:**
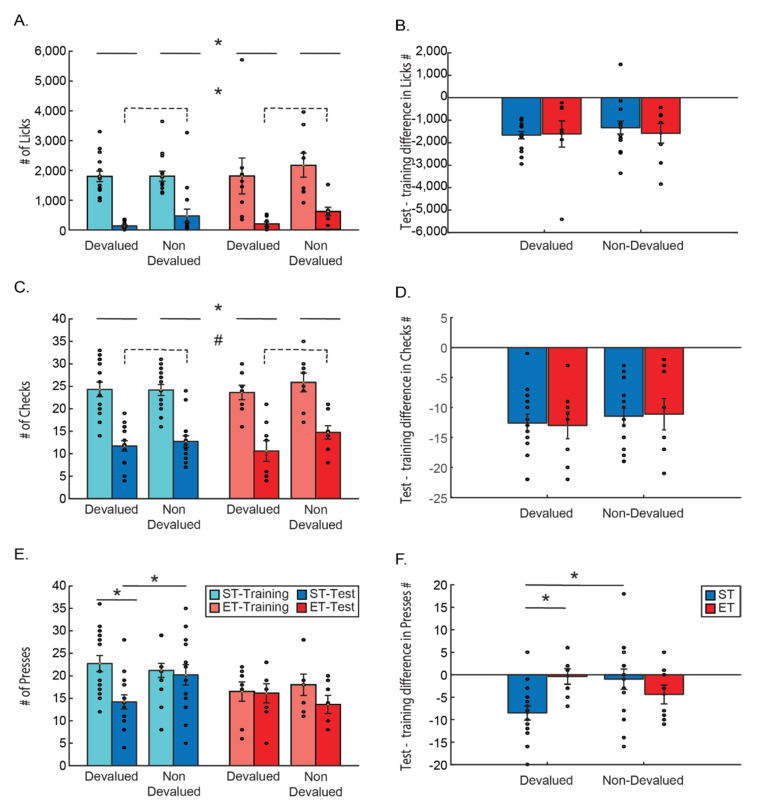
The task behaviors in the test following devaluation training. All following figures are bar plots with error bars (standard error) overlaid with the dots of raw data of each mouse. Blue represents the short training group (ST) and red represents the extended training group (ET). In each group, the lighter color represents the last day of training and the darker color—the test. Asterisk represents significance of *p* < 0.05. Hash (#) represents a statistical trend (0.1 > *p* > 0.05). Each figure on the left (**A**,**C**,**E**) shows the full comparison of each behavior between training groups, reward type, and time. Figures (**A**,**C**,**E**), show a comparison of the number of licks, magazine checks, and lever presses, correspondingly. In each figure, the data are shown for both the ST and ET groups, for the devalued and non-devalued rewards, and are presented separately for the last day of training and test sessions. Full lines represent the three-way ANOVA effects and dashed lines—two-way ANOVA effects. Figures (**B**,**D**,**F**) show the comparison of the differences (subtraction) between the test day and the last day of training in each mouse’s behavior between training groups and reward type. (**A**,**B**) presents the number of licks. (**C**,**D**) presents the number of magazine checks. (**E**,**F**) presents the number of lever presses.

Goal-directed behavior is more sensitive to change in reward value than habitually controlled behavior, which is more resistant to such a change. We therefore examined different behaviors during the task, looking for indications for sensitivity to the change in reward value caused by the devaluation. We found that while behaviors directly related to reward consumption (sometimes referred to as Pavlovian), such as magazine checks and licking, maintained sensitivity, regardless of the duration the training, the instrumental lever press behavior lost sensitivity to reward devaluation with extended training. Only in the lever press behavior, mice in the ET group did not show sensitivity to the reward value and continued to press both levers, while mice in the ST group attenuated devalued reward related behavior (Figure 2).

Each measured behavior was compared using a three-way ANOVA, to measure the changes as a function of condition (ST/ET), reward type (devalued/non-devalued), and time of measurement (last day of training/test). The last day of training is referred to as a baseline as it is the last day before the devaluation of one of the rewards, and as the test itself is under extinction conditions, no reward is present. The use of the last day of training as reference has led us to test each behavior by looking directly at the difference in behavior between the days, by subtracting for each mouse the behavior in the last day of training from the behavior in the test, and conducting a two-way ANOVA on the differences.

Both licking and checking behavior produced very similar results: the three-way ANOVA for the number of licks revealed no effect on licking other than the time (main effect for time F(1, 21) = 79.320, *p* < 0.001), with no other main effects or interaction of time with any of the other factors (all *p* > 0.1; n.s.; Figure 2A). We also found inhomogeneous variance between the groups compared in the three-way ANOVA. We therefore conducted a *t*-test with Welch–Satterthwaite degrees of freedom correction for the relevant planned comparison. This indeed revealed there were significantly more licks during the last day of training as compared to the test (t(45) = 10.64 *p* < 0.001). A mixed ANOVA looking at the difference between the number of licks in the test and the last day of training and comparing this difference between the type of reward (devalued/ non-devalued) and the training type (ST/ET) found no difference between the groups (ST/ET), no differences between reward types and no interaction (all *p* > 0.1; n.s.; Figure 2B), suggesting that there is no effect of the length of training or of devaluating the reward on this behavior. As the number of licks was immensely higher during training than in the test (due to the fact that the test was performed under extinction), comparing the number of licks during the test to the training could dilute any differences between devalued and non-devalued conditions during the day of the test. We therefore also conducted a two-way ANOVA between condition (ST/ET) and reward type (devalued/non-devalued) only during the test. We found a main effect for the reward, as there were significantly more licks to the non-devalued as compared to the devalued reward (F(1, 21) = 5.2951, *p* < 0.05). Yet, we found no difference between the different groups (F(1, 21) = 0.25996, *p* = 0.62 n.s.) or an interaction effect (F(1, 21) = 0.02746, *p* = 0.87 n.s.). This suggests that licking behavior was directly affected by the value of the reward or the lack of it, and this direct effect could not be changed due to prolonged training.

The three-way ANOVA for the number of magazine checks resembled the results of the licks and revealed no effect on checks other than the time of the session. A main effect for time F(1, 21) = 139.06, *p* < 0.001 was found with no other main effects or interaction of time with any of the other factors (all *p* > 0.1; n.s.; Figure 2C). This might suggest that the only factor affecting the checking behavior was the lack of reward, and not its value, which differentially changed only in one of the rewards. A mixed ANOVA looking at the difference between the number of checks in the test and the last day of training, and comparing this difference between the type of reward (devalued/ non-devalued) and the training type (ST/ET) found no difference between the groups (ST/ET), no differences between reward types and no interaction (all *p* > 0.1; n.s.; Figure 2B,D) suggesting that the length of training or the devaluation of the reward does not affect this behavior. Yet, in order to compare the lack of effects to the licking behavior, we also conducted a two-way ANOVA between condition (ST/ET) and reward type (devalued/non-devalued) only during the test. Here we found the same trend as the licking behavior. There was a trend for more checks to the non-devalued as compared to the devalued reward (F(1, 21)= 3.7526, *p* = 0.066). Yet, we found no difference between the different groups (F(1, 21) = 0.06913, *p* = 0.795 n.s.) or an interaction effect (F(1, 21) = 1.3952, *p* = 0.25073 n.s.). Like the licking behavior, this suggests that checking behavior could be affected by the value of the reward and is strongly affected by lack of it, and this direct effect could not be changed due to prolonged training.

The instrumental lever press behavior, however, is a different story. A three-way ANOVA comparing the number of presses by condition (ST/ET), time (last day of training/test), and lever associated with the reward type (devalued/non-devalued) revealed a main effect for time (F(1, 21) = 12.075, *p* < 0.01) with less presses on the test than in training. A main effect was also found for condition (F(1, 21) = 6.7639, *p* < 0.05) with less presses altogether of the ET group compared to the ST group. We also found a significant three way interaction: F(1, 21) = 6.6862, *p* < 0.05 (partial Eta^2^ = 24.2%; observed power of 69.4%) (Figure 2E). A Tukey HSD post hoc test revealed that a significant reduction in lever presses between the training and the test occurs only for ST mice (*p* < 0.01), and only for the lever associated with the devalued reward as compared to the non-devalued reward associated lever (*p* < 0.05). This suggests that the instrumental lever presses are affected specifically not from the lack of reward but from the value of the reward and only if the mice were shortly trained.

This effect was again evident when looking at the difference between the number of lever presses in the test as compared to the last day of training as the dependent variable (Figure 2F). A mixed ANOVA comparing the differences in licking between the type of reward (devalued/non-devalued) and the training type (ST/ET) found no main effects of either factors (both *p* > 0.1; n.s.). However, there was a significant interaction effect (F(1, 21) = 6.6862, *p* < 0.05) suggesting that the groups are different in the way they press the lever for the different rewards. A Tukey HSD post hoc test revealed that ST mice pressed the devalued reward related lever significantly less as compared to both the lever associated to the non-devalued reward (*p* < 0.05) and the number of presses on the devalued reward associated lever pressed by the ET group (*p* < 0.05). These results indicate again that the instrumental lever press behavior is affected by the devaluation of the associated reward only in shortly trained mice.

**Figure 3:**
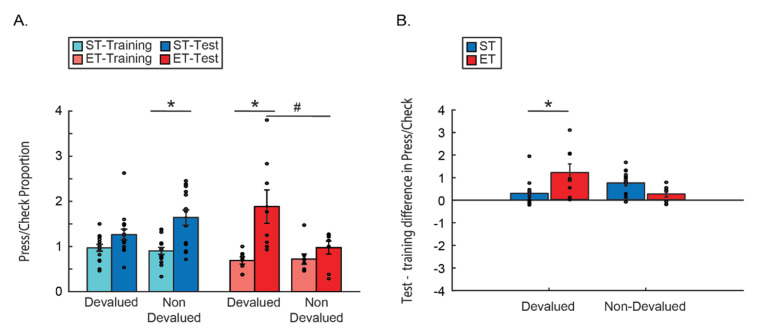
Lever press/magazine-checks proportions on the test following devaluation training. All following figures are bar plots with error bars (standard error) overlaid with the dots of raw data of each mouse. Blue represents the short training group (ST) and red represents the extended training group (ET). In each group the lighter color represents the last day of training and the darker color—the test. Asterisk represents significance of *p* < 0.05. Hash (#) represents a statistical trend (0.1 > *p* > 0.05). (**A**) Comparison of the proportions between training groups, reward type, and time. (**B**) Comparison of the difference (subtraction) between the test day and the last day of training in each mouse proportion between training groups and reward type.

As discussed in detail in the introduction, the different sensitivities of the different behaviors to devaluation after overtraining may lead to what seems like irrational behavior—excessive instrumental behavior without capitalizing on the effort and collecting the rewards. To test for this model behavior, we looked at the change in proportions between the instrumental lever press behavior and the more Pavlovian approach behavior. Proportion measure was computed by dividing the number of lever presses by the number of reward checks during the test for each mouse. Here indeed we found that ET mice worked much more (lever presses) and capitalize on their work much less (magazine checks) as their instrumental responses are maintained in the face of devaluation, yet their approach behavior maintains sensitivity to reward, and as such, declines (Figure 3). A three-way ANOVA comparing the press/checks proportions by condition (ST/ET), time (last day of training/test) and reward type (devalued/non-devalued) revealed a main effect for time (F(1, 21) = 49.797, *p* < 0.001),with no interaction of time with any of the other factors (all *p* > 0.1; n.s.). We also found a two-way interaction between reward type and condition (F(1, 21) = 5.9405, *p* < 0.05) and a three-way interaction: F(1, 21) = 10.722, *p* < 0.01. A Tukey HSD post hoc test revealed an increase in press/check proportions for the devalued reward only in the test after extended training—as compared to the last day of training of ET group (*p* < 0.005), and as compared to the response to the non-devalued reward in the ET group (*p* < 0.05). We also see an increase in press/check proportions for the non-devalued reward only in the test after short training—as compared to the last day of training of ST group (*p* < 0.01), but there is no difference from the proportion of their response to the devalued reward (*p* > 0.1 n.s.; Figure 3A). As Levene’s test showed that there is no homogeneity of the variances we conducted a *t*-test with Welch–Satterthwaite degrees of freedom correction for the relevant planned comparisons compared to a Bonferroni corrected alpha of 0.016. This revealed that the effects of time within the ET group and this effect in the ST group remains significant (t(8) = 3.18 *p* < 0.01; t(20) = 4.02 *p* < 0.01), while there remains a trend of the within ET comparison between the rewards (t(9) = 2.3 *p* < 0.02). Together with the main effect of time this shows a main tendency to press more and check less in the absence of reward. The effect of the extended training group however points to an added effect of the reduction in reward value, as ET mice lever press response becomes less dependent on the goal value while the checking behavior is still affected.

This effect was even more apparent when looking at the difference of this proportion between the test and the last day of training as the dependent variable (Figure 3B). A mixed ANOVA comparing the press/check proportions between the type of reward (devalued/non-devalued) and the training type (ST/ET) found no main effects of either factors (both *p* > 0.1; n.s.). However, there was a significant interaction effect F(1, 21) = 10.722, *p* < 0.005. Tukey HSD post hoc test showed that the press/check proportion increases only for the devalued reward and only for ET mice as compared to ST devalued reward (*p* < 0.05). This suggests that only the cumulative value of both the devaluation and the lack of reward in the test increased the difference in proportions between the instrumental lever presses and the Pavlovian approach, but only if mice were trained long enough for the lever press behavior to become more habitual and less controlled by the reward value.

#### 3.1.4. Revaluation

**Figure 4:**
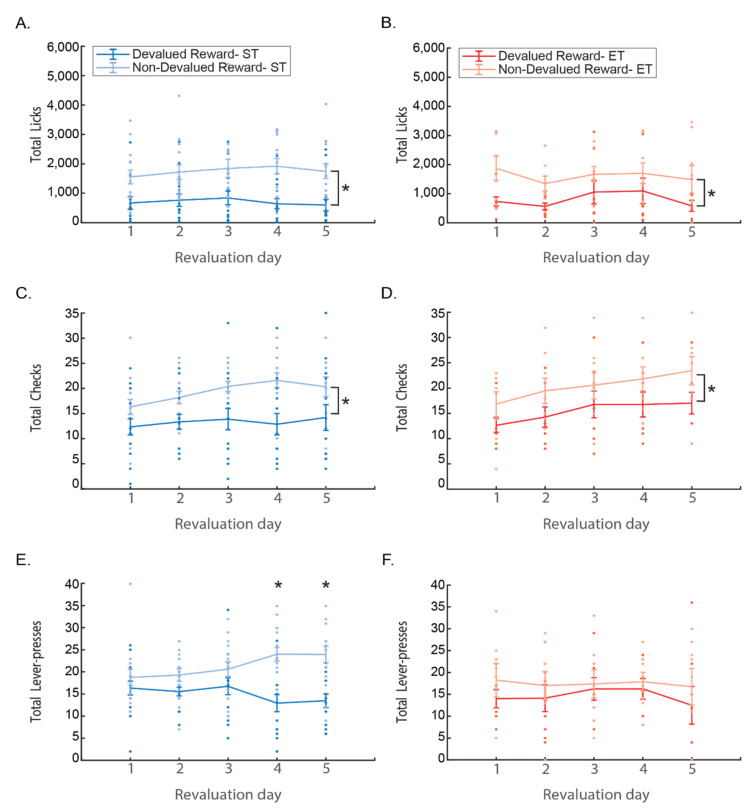
The task behaviors in the revaluation training. All following figures are line plots with error bars (standard error) overlaid with the dots of raw data of each mouse. Asterisk represents significance of *p* < 0.05. Blue (left figures **A**,**C**,**E**) represents the short training group (ST) and red (right figures **B**,**D**,**F**) represents the extended training group (ET). In each group, the lighter color represents the non-devalued reward and the darker color—the devalued reward; (**A**,**B**) presents the total number of licks; (**C**,**D**) presents the number of magazine checks; (**E**,**F**) presents the number of lever presses.

To test the persistence of devaluation effect on behavior, mice were trained for five extra days after the test session. The revaluation refers to the value of reward after the new value was acquired during devaluation. Both rewards were reinstated and no LiCl was injected, so mice could regain the value of reward. The reduced value of the reward persisted in both Pavlovian behaviors, as both licking and checking of the devalued reward were maintained at a lower level all through revaluation training for both ST and for ET mice. The instrumental lever press behavior, however, gives a strong indication to the different control mechanisms executing the behavior of each group. In the ST group, the difference between responses to the two rewards only grew with training, maintaining the difference in the value of the rewards acquired during devaluation as learning motivation. In the ET group, lever press behavior seemed to persist regardless of the reward value and was maintained through all revaluation days (Figure 4).

Comparing the number of licks in ST mice during the five days of revaluation training using a repeated two-way ANOVA with days and reward (devalued/non-devalued) as factors revealed a main effect only to reward type (F(1, 14) = 18.362, *p* < 0.001) with overall more licks to the non-devalued reward. No effect was found for days or any interaction effect (all *p* > 0.1 n.s.; Figure 4A). When looking at ET mice, the same two-way ANOVA reveals the same main effect only to reward (F(1, 7) = 9.8692, *p* < 0.05), with overall more licks to the non-devalued reward. Again, no effect was found for days or any interaction effect (all *p* > 0.1 n.s.; Figure 4B).

Magazine checks reveals the same tendency as licks both in ST and in ET mice. Repeated two-way ANOVA with days and reward (devalued/non-devalued) as factors revealing a main effect only to reward type F(1, 14) = 14.973, *p* < 0.005 with overall more magazine checks for the non-devalued reward with effect was found for days or any interaction effect (all *p* > 0.1 n.s.; Figure 4C). The same results was revealed in the ET mice where two-way ANOVA resulted in a main effect only to reward F(1, 7) = 13.629, *p* < 0.01, with overall more licks to the non-devalued reward. Here, a trend effect was found for days F(4, 28) = 2.5417, *p* = 0.06 with Tukey post hoc comparison showing that checking in the last day tends to differ from the first day (*p* = 0.06), but without any interaction effect (*p* > 0.1; n.s.) suggesting this increase is the same for both rewards (Figure 4D).

Here, also, the instrumental lever press behavior was different than both licking and checking. Comparing the number of lever presses of the ST mice during the five days of revaluation training using a repeated two-way ANOVA with days and reward (devalued/non-devalued) as factors revealed a main effect of reward type (F(1, 14) = 9.6156, *p* < 0.01), with overall more lever presses to the non-devalued reward. While no main effect was found for days (*p* > 0.1 n.s.), there was a significant days * reward interaction effect (F(4, 56) = 4.3003, *p* < 0.005), with Tukey Post hoc test showing that the difference in lever presses between devalued and non-devalued rewards started from the 4th day and continued to the last day (difference between reward types in 4th and 5th days all *p* < 0.001). These results suggest that the negative value acquired during devaluation continued and even got stronger when re-exposed to the rewards (Figure 4E). When looking at ET mice, the same two-way ANOVA shows no difference in either reward, days, or any interaction between them (all *p* > 0.1; n.s.). Implying that due to extended training the lever pressing behavior is no longer goal-directed and the value of the reward either from re-exposure to the reward or from devaluation does not affect the number of lever presses after extensive training (Figure 4F).

## 4. Discussion

The nature of compulsions and neural evidence has led to a theory that OCD stems from a dysfunctional communication between two systems for action control—the habitual and the goal-directed behavior systems [2]. The first promotes automation of selected behavior that occurs implicitly and the second promotes conscious and explicit information processing. In this research, we set to establish a behavioral mouse model enabling the differentiation between the different action control strategies applied to different kinds of learned behaviors. The task associates two different levers with two different rewards. We used overtraining of goal-directed lever press behavior to reach habitual control and a devaluation process to reduce the value of one of the rewards and test for action control.

The results of the training show that all measured behaviors (licking, checking, and lever pressing) are increased during the training. This, together with the increase in the proportions of correct lever presses, indicates that mice learned the task already during the short phase of the training, and high performance was maintained during extended training. The devaluation training results indicate that we were able to reduce the value of only one of the rewards to a significant extent both for the ST and for the ET mice. Overall, these results indicate that the test was conducted on mice that successfully learned the task. That is, which is the correct lever to press following the specific cue, what reward it would yield, and the value of that reward. Therefore, the significant differences in post-devaluation lever press performance between the ET and ST groups indicate the difference in the ability of the reward value to affect behavior due to the amount of training.

We found that unlike the over-trained mice, which maintained high numbers of lever presses for both rewards, the ST group significantly decreased the lever presses to the devalued reward, both compared to the last day of training and to the presses during the test for the non-devalued reward, suggesting that prolonged training rendered lever press actions less dependent on reward value and more stimulus–response driven. This is even more interesting when considering the ET group showed significantly less lever presses in general (taking together the last day of training and the test), which might suggest that this behavior became more stimulus–response and less goal dependent already in the late phases of overtraining. This, however, was not the case, for the licking behavior or for the magazine checking behavior. While it seems that the reward value change due to devaluation does influence those behaviors, this effect does not seem to be affected by the amount of training. This reduction in behavior due to absence of the reward in the test could suggest that those behaviors are highly and directly sensitive to the reward, and the lack of it had a direct and much more pronounced effect than the newly learned reward value. The sensitivity to the change in reward value was, however, much more noticeable in the results of the revaluation days where reward was returned to the arena. The presence of the now differentially valued rewards maintained both licking and checking under lower performance for the devalued than for non-devalued rewards, without considering the length of the training. The revaluation has also highlighted the difference in sensitivity to reward of the instrumental lever press behavior as the difference in performance to the lever associated to the devalued vs. non-devalued reward grew with training, but only in the ST group. In the ET group, the similar performance on both levers was maintained. This further indicates that, while the action of lever pressing associated with the sound became habitual for the mice through extensive training, both licking and checking behaviors were less prone to such a change.

Magazine approach or checking was shown, before, to be resistant to becoming habitual [23,24]. Here, we also show the resemblance in this behavior to the licking behavior. Licking is a measure of direct taste reactivity, and was also shown to be affected by conditioned taste aversion [29]. The fact that magazine checking reacts both to the lack of reward and to the devaluation, in the same way, licking implies that this behavior is closer to this direct reward reaction than to the instrumentally learned lever press action. Instrumental lever presses on the other hand was shown to be independent of the value of reward [30], especially after prolonged training [17,20]. It was suggested that the response-outcome (R–O) association underlying the instrumental response in its forging, gradually changes with training to a stimulus–response association [17]. Therefore, prolonged training has a differential effect on lever presses, which becomes less dependent on outcome value as compared to magazine activity, which maintains direct outcome value control [31]. It was further suggested that those processes are sustained by different psychological [32] and neural processes [31].

The classical PTSA rat model demonstrates excessive lever presses behavior unfollowed by checking behavior, after the contingency between the stimulus and the reward has been extinguished [12]. This behavior was found to be sensitive to drugs and treatments effective for alleviating compulsive symptoms [13,14,15], suggesting perhaps some overlap between learning mechanisms underlying this phenomena and the neural mechanisms of compulsive behavior [16]. Even though our method of changing the outcome value differs from the original PTSA model, we did find somewhat similar behavior in our model. When looking at the proportions between the number of presses and the number of checks we found an increase in proportions (more presses vs. less checks), but only for the lever/magazine associated with the devalued reward, and only for mice that were extensively trained (ET). This suggests that the high and direct sensitivity of the approach behavior to the value of the absent (during the test) and devalued reward has attenuated this behavior while the lever press behavior which acquired S–R control due to the prolonged training was maintained at high levels. This discrepancy in the control mechanism could have created what appears as excessive and maladaptive instrumental behavior. However, this does not make the lever press behavior after extended training, compulsive, but stresses the difference in the flexibility of different behaviors, which on the extreme could potentially help in the manifestation of OCD.

In our model, we add another layer, looking at the different behaviors when the mice can resample the two rewards in the revaluation training. We find that, not only does the effects on licking, checking, and lever-pressing are maintained, and that the difference between them persists, but it even gets stronger. As mice are re-experiencing the rewards, now with their renewed values, licking and checking behaviors are attenuated for the devalued reward both in ST and ET mice, while the difference in lever press behavior to the two rewards increases with training for the ST group, but remains the same all through training for the ET group. It seems that once the control of the lever press behavior shifted towards S–R, it remains persistently unchanged.

One significant limitation of this research is that it lacks an examination of the neurobiological correlates associated with the described behaviors. Both the habitual and the goal-directed behavioral control systems, which are tested by our task, were well described to be mediated by parallel basal–ganglia–thalamocortical circuits [33]. Each circuit is organized as a direct excitatory pathway, maintained under control of an indirect inhibitory pathway; a configuration that allows for the selective inhibition/disinhibition of each circuits output [33,34,35]. The parallel loops theoretically funnel functionally different information and include, among others, the limbic loop, which processes motivational, stimulus–outcome information, the associative loop, involved in action–outcome and, therefore, goal-directed control, and the sensory motor loop that mediates stimulus–response behaviors and, therefore, habit control [2,36,37]. The interconnections between these loops [38,39] creates a hierarchy of information transfer and provide a potentially efficient action selection mechanism [40]. The ability to change between these two strategies of behavioral control is essential for adaptive behavior, and the failure to do so was suggested to lie at the root of pathologies, such as OCD [2,4,5,6]. As some of the behaviors in our task seem to transition from goal-directed to habit with extensive training while other behaviors maintain their goal-directed control even after extensive training, localizing the regions and circuits involved in this phenomenon could add strength to our results and help pinpointing the physiological origins of our results. Hence, though through a unique comparison of several levels of behavior towards different rewards of different values as they change through training and time, this research provides a valid framework for future testing of the neural correlates of changes in behavioral control.

As mentioned earlier, a theoretical framework of studying OCD suggest that compulsive behavior could be manifested by a faulty goal-achieving signal, or a deficit in the feedback associated with the appropriate performance of goal-directed responses [10,11,41,42,43,44,45,46]. In our model, we find that the ability of a behavior, especially instrumental behavior, to adapt to a change in circumstances (a change in the value of a known reward), depends on the flexibility of the system in control of the behavior. That is, once an action was practiced extensively it becomes habitual and, thus, under S–R control, and could not be amended, not even when resulting in a noxious outcome. Unlike the instrumental behavior, behavior directly aimed at the outcome, such as licking or checking, responds immediately to the change in value. One could consider an imbalance or an impairment in the balance between the systems rendering a behavior excessive and unresponsive to changes in outcome while experiencing the change as something that should have changed behavior.

## 5. Conclusions

Different behaviors preformed by mice in an appetitive lever-press task, namely licking, checking and lever-press behaviors, show different sensitivity to reward devaluation after extensive training. While both licking and checking behavior maintain sensitivity to reward value, instrumental lever-press behavior becomes insensitive after prolonged training. This difference is maintained days after the devaluation procedure, and after re-experiencing the reward without the noxious consequences during revaluation days. The results of the current study show that different behaviors are maintained under different mechanisms of action control with different flexibility in response to changes. This usually functional system could potentially when out of balance create unresolved discrepancies between excessive non adaptive behaviors and a well acknowledged change.

## Data Availability

The data collected in this study are available from the corresponding author upon reasonable request.
